# Airborne Bacteria in CAFOs: Transfer of Resistance from Animals to Humans

**Published:** 2005-02

**Authors:** Julia R. Barrett

Antibiotics are used in concentrated animal feeding operations (CAFOs) to treat and prevent livestock disease and to bolster animal growth and the nourishment efficiency of feed. These nontherapeutic uses involve long-term, low-level dosing that creates an appropriate environment for bacteria to develop antibiotic resistance. Several antibiotics used in animal agriculture are the same as or similar to those used in human medicine; transference of resistant microbes from animals to humans could further undermine antibiotic effectiveness against human disease. A research team including Amy Chapin of the Johns Hopkins Bloomberg School of Public Health examines one possible way that resistance may be transferred from animals to humans **[*EHP* 113:137–142]**.

Previous studies have examined the potential for infection with resistant microbes via animal waste–polluted water in the vicinity of a CAFO and contaminated food products. This study offers evidence for infection occurring in a way that has not been previously considered.

According to the Johns Hopkins team, inhalation of airborne bacteria could constitute another exposure pathway. It is already well documented that air within swine CAFOs can be heavily contaminated with bacteria. Several of the bacterial species are normally present in animals and humans but can sometimes cause illness. The current study is one of the first to investigate antibiotic resistance in airborne bacteria in a swine CAFO.

Working in a swine finishing CAFO in the mid-Atlantic United States, the researchers collected air samples in December 2003 and January 2004. The air samples were then conveyed to the laboratory for bacterial isolation and speciation. Initial tests yielded 137 presumptive *Enterococcus* species isolates, and further tests confirmed that 47 were enterococci. Of the remaining 90 isolates, 44 were coagulase-negative staphylococci, 45 were viridans group streptococci, and 1 was *Micrococcus luteus*.

Each isolate then underwent testing to determine susceptibility to the antibiotics erythromycin, clindamycin, tetracycline, vancomycin, and virginiamycin. The first four of these drugs are used in human medicine; the last, virginiamycin, closely enough resembles a human drug that bacteria resistant to one will be resistant to the other. Of the five antibiotics, only vancomycin is not approved for livestock use in the United States.

All of the isolates were susceptible to vancomycin, but 121 were resistant to at least two of the antibiotics used in swine production; 115 were resistant to three. These results underscore the relationship between antibiotic use and the emergence of resistance: in the absence of use, resistance is unlikely to develop.

In some situations, the bacterial species found in CAFO air samples can cause human disease. *Enterococcus* species and coagulase-negative staphylococci are leading causes of infections in health care settings. Viridans group streptococci, normally found in the respiratory tract, are linked to life-threatening infections in immune-compromised individuals. The viridans group streptococci are also suspected reservoirs for erythromycin resistance genes, which could potentially be transferred to more pathogenic streptococci.

The researchers conclude that exposure to airborne bacteria from a CAFO presents a potential pathway for transferring antibiotic-resistant bacteria from animals to humans. CAFO workers and the people with whom they come in direct contact, as well as neighbors near the operations and areas of land where animal wastes are applied, may be especially at risk. Continuing research of the transfer of antibiotic-resistant bacteria from animals to humans needs to encompass a variety of environmental media that may serve as exposure sources.

## Figures and Tables

**Figure:**
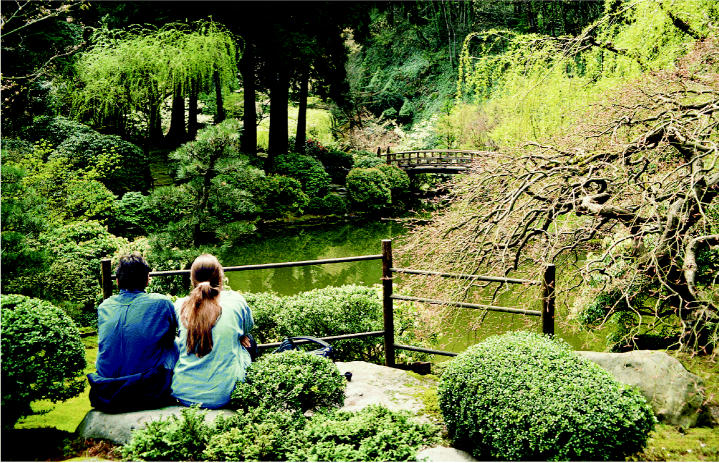
**Building and bonding.** Understanding the stress-fighting effects of certain elements of the built environment may shed light on how people’s surroundings affect their quality of life.

